# Steroidogenic Acute Regulatory Protein in Breast Cancer: Mechanistic Insights into Pathogenesis and Therapeutics

**DOI:** 10.3390/ijms27073117

**Published:** 2026-03-30

**Authors:** Arpita Marick, Britney Manna, Hafiz Khan, Pulak R. Manna

**Affiliations:** 1 Department of Internal Medicine, Texas Tech University Health Sciences Center, School of Medicine, Lubbock, TX 79430, USA; arpita.marick@ttuhsc.edu; 2 Department of Biology and Biochemistry, Texas Tech University, Lubbock, TX 79409, USA; bmanna@ttu.edu; 3 Covenant Medical Health Systems, Covenant Hospital, Lubbock, TX 79410, USA; 4 Department of Public Health, Texas Tech University Health Sciences Center, Julia Jones Matthews School of Population and Public Health, Lubbock, TX 79430, USA; hafiz.khan@ttuhsc.edu

**Keywords:** breast cancer, StAR, aromatase, estrogen overproduction, aromatase inhibitors, HDAC inhibitors, therapeutic strategy

## Abstract

Breast cancer (BC), a multifactorial condition, remains one of the most common malignancies in women, in which the majority of BCs are hormone-sensitive and are activated by estrogens, especially 17β-estradiol (E2). Whereas aromatization of androgens to estrogens is achieved by the aromatase enzyme, the steroidogenic acute regulatory (StAR) protein, by mobilizing the transport of intra-mitochondrial cholesterol, plays an indispensable role in E2 biosynthesis. Accumulating evidence indicates that aromatase expression is aberrantly high and analogous in normal and malignant breast tissues, even though endocrine therapy, based on aromatase inhibitors (AIs), has been the mainstay of BC treatment in post-menopausal women. Despite the beneficial effects of AIs, their long-term usage has been associated with undesirable long-term side effects, including endocrine resistance, which is the leading cause of cancer death, warranting an improved therapy for mitigating this devastating disease. Along these lines, we reported that StAR is differentially expressed, along with E2 biosynthesis, in human and mouse cancerous and non-cancerous breast cells and tissues, in which we discovered that StAR is an acetylated protein, in addition to the identification of a number of lysine residues, undergoing acetylation and deacetylation, suggesting the importance of this newly uncovered StAR modification in E2 regulation in mammary tissue. One of the current therapeutic approaches for BC is targeting with histone deacetylase inhibitors (HDACIs), as these epigenetic enzymes control multiple cellular processes, including chromatin remodeling and genomic stability through the dynamic process of acetylation and deacetylation of core histones. Concomitantly, we have demonstrated that several HDACIs, including FDA-approved HDACIs, at therapeutically and clinically relevant doses, alter StAR acetylation patterns and suppress E2 accumulation in both hormone-sensitive human BC and mouse primary cultures of breast tumor epithelial cells. This review provides the molecular insights into breast pathogenesis and its therapeutics, and proposes that a combination therapy involving AI and HDACI, targeting aromatase and StAR, respectively, suppresses intra-tumoral E2 accumulation and limits antagonistic side effects, and these measures are beneficial for the prevention and/or management of hormone-sensitive BC.

## 1. Introduction

The steroidogenic acute regulatory (StAR, commonly known as StAR-related lipid transfer domain-containing protein 1, STARD1) protein mediates the rate-limiting step in steroid biosynthesis; thus, it plays an important role in diverse steroid-led physiological and pathophysiological processes [1,2,3,4]. StAR has a 37-kDa precursor form containing an NH_2_-terminal mitochondrial targeting sequence that is utilized by the STARD1-6 family of proteins and a COOH-terminal cholesterol-binding StAR-related lipid transfer (START) domain [5,6,7,8,9]. It comprises a ~7.5 kb gene that includes seven exons and six introns encoding 284 and 285 amino acids in mouse and human, respectively. The COOH-terminal region of StAR is critical for regulating the steroidogenic response, as truncation/mutation of relevant amino acids markedly decreases the biological activity of StAR. Tissue-specific regulation of StAR, and thus steroid hormones, is influenced by a plethora of signaling pathways and involves transcription, translation, or activation of this cholesterol transporter. Virtually all studies, to date, have shown that agents and/or factors that influence StAR also influence the synthesis of steroids via endocrine, autocrine, or paracrine mechanisms [5,6,7,8,9]. Along these lines, we have discovered a post-translational modification (PTM) of StAR, acetylation, and this alteration is capable of overproducing estrogens, especially 17β-estradiol (E2), in breast cancer (BC) cells, which promotes breast tumorigenesis [2,8].

BC is the second leading cause of cancer-related mortality among women worldwide. In 2025, the United States is projected to report 316,950 new BC cases and 42,170 related deaths, accounting for ~12% of all recent cancer cases [10,11,12]. BCs are broadly classified into hormone receptor-positive (HR+) and hormone receptor-negative (HR-) subtypes [1,13]. HR+ BCs are characterized by the expression of estrogen receptor (ER), progesterone receptor (PR) or both, and may express human epidermal growth factor receptor 2 (HER2) [14,15]. Notably, hormone-dependent BCs constitute ≥80% of all BC cases, involving luminal A and luminal B subtypes that are largely dependent on E2 activation, which is the principal driver of tumor initiation, growth, and progression [15]. In pre-menopausal women, the ovaries are the primary source of E2; however, following menopause, estrogen production occurs mainly in peripheral tissues, including adipose and skin [13,16]. HR+ BC in post-menopausal women is strongly associated with obesity, which alters/elevates insulin, adipose-derived E2, and inflammatory mediators, indicating dysregulation of cellular mechanisms for promoting uncontrolled growth of breast epithelial cells [17,18,19]. Furthermore, dysregulation of estrogen biosynthesis, especially E2, modulates genetic and epigenetic signaling, and promotes the growth and maintenance of hormone-sensitive BC [1,2,13,20,21,22]. BC cells/tissues frequently accumulate elevated levels of cholesterol and/or its derivatives, associated with enhanced tumor aggressiveness and cellular proliferation [23]. Accordingly, 27-hydroxycholesterol has been implicated in the progression of HR+ BC [24]. Estrogen/E2 biosynthesis is regulated by the aromatase enzyme, encoded by the *CYP19A1* gene, which catalyzes the conversion of androgens to estrogens, and these events represent a key determinant for local estrogen availability [25]. Even so, recent studies have reported that aromatase expression is analogous not only in HR+ and HR-BC subtypes, but also in non-malignant breast cells and tissues [2,4,26,27,28,29], underscoring that an aromatase catalyzed event may not be crucial in E2 regulation in mammary tissue.

Endocrine therapies targeting ER or E2 are beneficial for managing HR+ BC. Hormone-sensitive BC is often treated with a variety of drugs that modulate ER or reduce E2 levels [15]. Of note, aromatase inhibitors (AIs) have been frequently used in the clinics for BC treatment in post-menopausal women [30,31]. Specifically, third-generation AIs, i.e., Anastrozole, Letrozole, and Exemestane, have become the standard of care in this population, administered either as monotherapy or in sequential regimens to enhance clinical outcomes [32]. While AIs effectively suppress systemic estrogen/E2 biosynthesis, their use is associated with many adverse long-term side effects, including bone mineral density loss, osteoporosis, arthralgia, and increased fracture risk. Notably, resistance to AIs remains a major clinical obstacle that contributes to BC mortality [4,12,32]. BC therapies encompass systemic and targeted treatments that are contingent on tumor subtypes and stages. However, increasing therapeutic intensity is accompanied by substantial toxicity and evidence that anticancer treatments can perturb genomic and epigenetic networks, contributing to disease progression and resistance [33,34]. Epigenetic dysregulation is a primary event in breast tumorigenesis. Histone deacetylases (HDACs) are a family of epigenetic enzymes, in which DNA methyltransferase (DNMT), histone acetyltransferase (HAT), and histone methyltransferase inhibitors have emerged as promising therapeutic interventions [35]. HDACs are frequently dysregulated in BC and other cancers [36]. It is noteworthy that HDAC inhibitors (HDACIs) show anti-proliferative and DNA repair–modulating effects in preclinical BC models [35,36]. Importantly, the majority of clinical trials investigate HDACIs in combination with other treatments tailored to specific molecular subtypes of BC [37]. Consequently, we reported that a number of HDACIs, including the FDA-approved ones, at clinically relevant concentrations, modulate acetylation of StAR and concurrently attenuate E2 levels in HR+ BC cells [2]. Of note, StAR is abundantly expressed, along with E2 synthesis, in various hormone-dependent human BC cell lines and mouse models of spontaneous breast tumors, compared with little to no StAR and E2 levels in their hormone-independent counterparts, signifying tumor-promoting and diagnostic relevance of StAR in BC [2,4]. Alterations in StAR, involving acetylation, can augment E2 overproduction for promoting breast pathogenesis. As a consequence, HDACIs have been shown to attenuate E2 accumulation in BC cells by influencing StAR acetylation [4,13]. Given the multifactorial nature of BC and the persistent risk of adverse side effects as well as recurrence, it is plausible that a combinatorial approach employing suboptimal doses of AI and HDACI may be more effective to target the disease pathogenesis while minimizing treatment-related toxicity for better outcomes.

## 2. Activation of Estrogen Signaling and Its Correlation to Breast Pathogenesis

Estrogens are a group of steroid hormones that are predominantly produced by the ovaries [38]. E2 is the most important candidate for influencing diverse physiological activities [39,40]. After menopause, estrogen/E2 is primarily synthesized by peripheral conversion of androstenedione and testosterone [41]. Estrogen biosynthesis begins with androgens, which are converted to estrogens by the aromatase (*CYP19A1*) enzyme [42,43,44]. Several studies have reported that E2 levels in stage-specific BCs can increase up to 30 times higher than those either in circulation and/or their normal counterparts [4,45,46,47]. Estrogens exert their effects through both genomic and non-genomic pathways. Genomic signaling occurs via direct and indirect mechanisms. Classically, estrogen binds to its receptors: ERα (*ESR1*) and ERβ (*ESR2*), triggering their dimerization and translocation into the nucleus. The receptor complex then regulates the transcription of estrogen-responsive genes by binding to estrogen response elements and/or interacting with other transcriptional regulators [40,48,49]. It has been reported that a number of signaling pathways, including ERα, HER, and mitogen-activated protein kinase (MAPK), interact to facilitate E2 overproduction in activating breast tumorigenesis [50,51]. In contrast, indirect genomic signaling, often referred to as transcriptional crosstalk, occurs without direct DNA binding. Instead, ER forms complexes with other transcription factors and response elements. For example, ERs can interact with the transcription factor specificity protein-1, enhancing its binding to GC-rich promoter regions and thereby influencing gene expression [48]. In addition to these genomic signaling, estrogens also induce rapid non-genomic responses. Although structurally identical to cytoplasmic ERα, membrane-localized ERα (mERα) differs in function. Upon ligand binding, mERα homodimerizes and activates the Gα and Gβγ subunits of heterotrimeric G proteins, leading to elevated intracellular cAMP and calcium levels [52]. Activated mERα interacts with various proteins, including the p85 regulatory subunit of phosphoinositide 3-kinase (PI3K) and the SH2 domain of c-Src kinase, and can associate with epidermal growth factor receptor. These interactions modulate downstream cascades, including extracellular signal-regulated kinase, MAPK, and PI3K/AKT signaling, which promote cell proliferation, survival, and migration, key events in BC progression [50,52].

**Figure 1:**
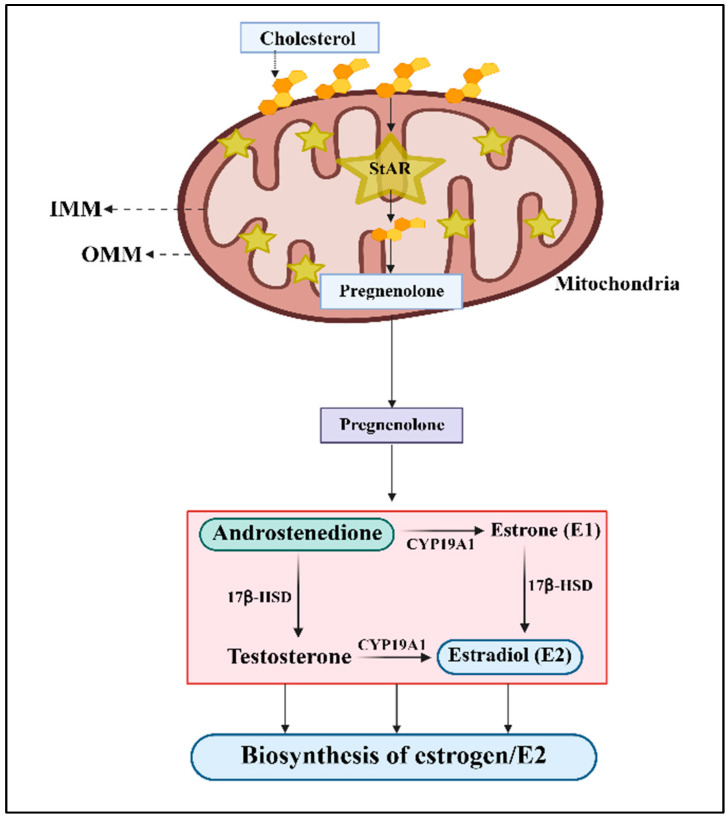
The steroid biosynthetic pathway that leads to the synthesis of estrogens in peripheral and/or breast tumor tissues. Cholesterol is transported into the IMM by the StAR protein, in which CYP11A1 converts cholesterol to pregnenolone, which is further processed through enzymatic steps to produce androstenedione. Aromatase is the key enzyme in estrogen biosynthesis, facilitating the conversion of androgens to estrogen/E2.

Regardless of the mechanisms involved, the transport of cholesterol from the outer mitochondrial membrane (OMM) to the inner mitochondrial membrane (IMM) has been primarily regulated by the mitochondrial StAR protein (Figure 1); thus, it plays an important role in E2 biosynthesis [6,8,9,53,54]. Pregnenolone is the first steroid produced at the IMM by the action of the cytochrome P450scc enzyme (CYP11A1), which exits the mitochondria and is then converted to various steroid hormones by a series of tissue-specific enzymes. Once androgens are produced in the steroid biosynthetic pathway, aromatase catalyzes the conversion of androstenedione and testosterone to estrogen/E2, in which the 17β-HSD (17β-hydroxy steroid dehydrogenase) enzyme plays an important role. It has been demonstrated that upregulation of 17β-HSD positively correlates with ER and promotes HR+ BC [55], suggesting the importance of aromatase and 17β-HSD in E2 biosynthesis in mammary tissue.

### 2.1. The Aromatase Enzyme and Estrogen Regulation

Aromatase, a cytochrome P450 mono-oxygenase, catalyzes the conversion of C19 androgens, including testosterone, into C18 estrogens such as E1 and E2, through three sequential oxidative steps that require oxygen and NADPH as co-substrates [43,56,57]. This occurs predominantly in breast and adipose tissues, where aromatase is highly expressed [44,58]. Aromatization takes place in the endoplasmic reticulum, and it is required to maintain estrogen levels, especially in post-menopausal women [46,57]. The levels of aromatase and other enzymes involved in estrogen synthesis are not consistent across different tissues, but instead depend on the local cellular environment and specific cell types, such as glandular or stromal cells [45,46]. This supports the notion that aromatase regulation in breast tissue is context-dependent, with differences between normal and cancerous cells reflecting the use of alternative promoters [59]. As mentioned above, aromatase expression does not correspond to estrogen/E2 biosynthesis, emphasizing the involvement of an alternate mechanism in E2 regulation in mammary tissue.

Transcriptional regulation of the *CYP19A1* gene is mediated by ten alternatively spliced promoters that are differentially utilized in normal and malignant breast tissues [1,45]. A switch of aromatase promoter usage from I.4 to I.3/II drives elevated E2 production in fibroblast adjacent to malignant epithelial cells [45]. Of significance, aromatase is acetylated on multiple lysine residues in BC cells, and additional acetylation sites emerge when Sirtuin (SIRT) deacetylase activity is inhibited. These acetylation marks cluster in the promoter regions important for substrate binding and catalytic function, indicating that aromatase acetylation directly modulates the enzyme’s ability to convert androgens to estrogens in BC cells [60]. In addition, the functional activity of aromatase can be elevated by cytokines and growth factors, boosting local estrogen production and promoting the growth of HR+ tumors [61].

### 2.2. Role of the StAR Protein in E2 Biosynthesis

The StAR protein mediates the rate-limiting step in steroid biosynthesis. Regulation of the StAR protein is predominantly influenced by cyclic adenosine monophosphate (cAMP) through activation of the protein kinase A (PKA) pathway, in which a plethora of processes play permissible roles [8,62,63]. Additionally, regulation of the StAR gene is highly complex, which consists of numerous transcription factors binding to distinct and overlapping regulatory elements concentrated within a short promoter region immediately upstream of the transcription start site [54,63]. Importantly, loss-of-function of StAR, involving mutations, leads to an inactive protein that markedly impairs steroid hormones, resulting in a lethal disease called lipoid congenital adrenal hyperplasia [64]. Thus, precise regulation of StAR at the transcriptional, post-transcriptional, and post-translational levels is crucial for preserving steroid hormone balance in maintaining bodily homeostasis and reproductive capacity [8,54,62,63].

It is unequivocal that sustained exposure to elevated estrogen levels, along with associated genotoxic and proliferative signaling, contributes to the initiation, survival, and growth of hormone-sensitive BC. Epidemiological risk factors such as menopause, family history, and extended use of oral contraceptives further underscore the importance of estrogenic stimulation in disease development and progression [1,6,8,65,66]. Emerging evidence indicates that epigenetic regulators modulate the expression and activity of both StAR and aromatase, thereby influencing E2 overproduction for promoting breast pathogenesis [4,8,28]. In support of this, analyses of RNA-seq data from The Cancer Genome Atlas breast invasive carcinoma (TCGA-BRCA) have demonstrated that amplification of the StAR gene, but not other steroidogenic enzymes, including aromatase, is associated with poor survival of BC patients [3,8]. These findings suggest that cholesterol transport mediated by StAR, rather than aromatase activity, represents a critical regulatory step in additional estrogen/E2 production within breast tumors [67,68]. Alternatively, StAR overexpression has been observed in hormone-responsive BC cell lines and transgenic mouse models of spontaneous breast tumors, compared to their non-tumorous counterparts, correlating with increased E2 synthesis, events that are consistent with tumor–node–metastasis (TNM) stages [3,8]. Although phosphorylation is known to enhance StAR activity for optimal steroidogenesis, we reported that StAR was not found to be phosphorylated in BC cells, rather acetylated, highlighting that StAR acetylation is a critical event for E2-activated breast tumorigenesis [8,13]. In this connection, the involvement of the STARD family proteins, especially STARD3-6, with significant homology to StAR/STARD1, in estrogen/E2-responsive BC, cannot be excluded [3,5,69,70]. Noteworthy, STARD3, a late endosomal protein with ∼37% homology to the COOH-terminus of StAR, was initially identified and cloned as a gene amplified in HER+ BC [71,72,73].

## 3. Acetylation of StAR and Its Relevance to E2 Overproduction and Breast Pathogenesis

Acetylation is a highly dynamic and reversible PTM that regulates protein activity and cellular function by influencing chromatin architecture and signaling pathways, which impact diverse biological processes, including cancers [8,13]. A well-studied example is histone acetylation, where acetyl groups are added to lysine residues on histones H3 and H4, a reaction catalyzed by histone acetyltransferases (HATs), also called ‘writers’ [74]. Histone acetylation plays a direct role in cAMP-stimulated steroid hormone production, loosening chromatin structure and making DNA more accessible for transcription [75]. Conversely, removal of acetyl groups, also known as ‘erasers’, leads to a more condensed chromatin state and transcriptional repression. Studies have shown that inhibition of HDAC activity with Trichostatin A (TSA) has been shown to increase StAR expression and enhance steroid biosynthesis [74,76]. In addition, TSA suppresses endothelial cell proliferation by modulating signal transducer and activator of transcription 5A-dependent transcriptional activity [77]. This repression downregulates a number of cell cycle-associated genes, including cyclin-dependent kinase (CDK) regulatory subunit 1B, an event that is beneficial for preventing BC proliferation [77].

**Figure 2:**
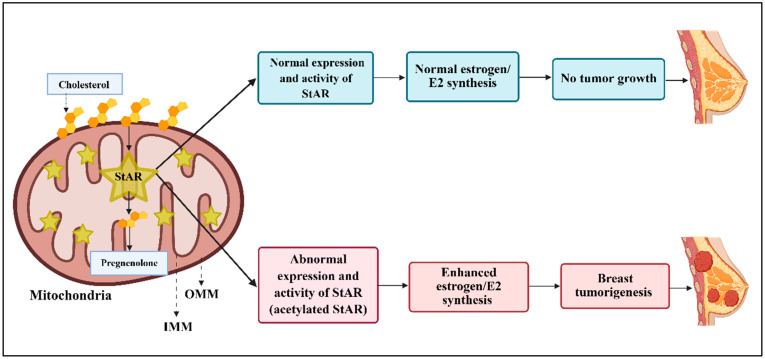
A schematic representation depicting diverse expression and activity of StAR in E2 biosynthesis. Under normal conditions, StAR-mediated E2 production maintains appropriate breast development and function (upper panels). In contrast, acetylation of StAR (bottom panels) enhances its biological activity, leading to increased E2 production, which promotes breast tumorigenesis.

PTMs play crucial roles in protein functions, protein–protein/DNA interactions, as well as their stabilities, in which the association of disease-specific modifications is paramount for the development of potential biomarkers and relevant therapies [13,60,78,79]. There is increasing evidence that lysine (K) acetylation sites in the majority of the mitochondrial proteins and peptides exhibit both stimulatory and inhibitory effects on their functional activities [80,81]. While mitochondrial acetylation sites are frequently controlled by the enzymatic activity, reversible protein acetylation has been critical in understanding its dynamics, impacting a variety of cellular functions. Along these lines, acetylation of StAR results in an increase in E2 overproduction, a crucial event in the progression and survival of BC. Analyses of BC and non-cancer breast cell lines have revealed a strong correlation between StAR expression and resultant E2 biosynthesis, suggesting that acetylation serves as a tumor promoter in this life-threatening disease (Figure 2).

**Figure 3:**
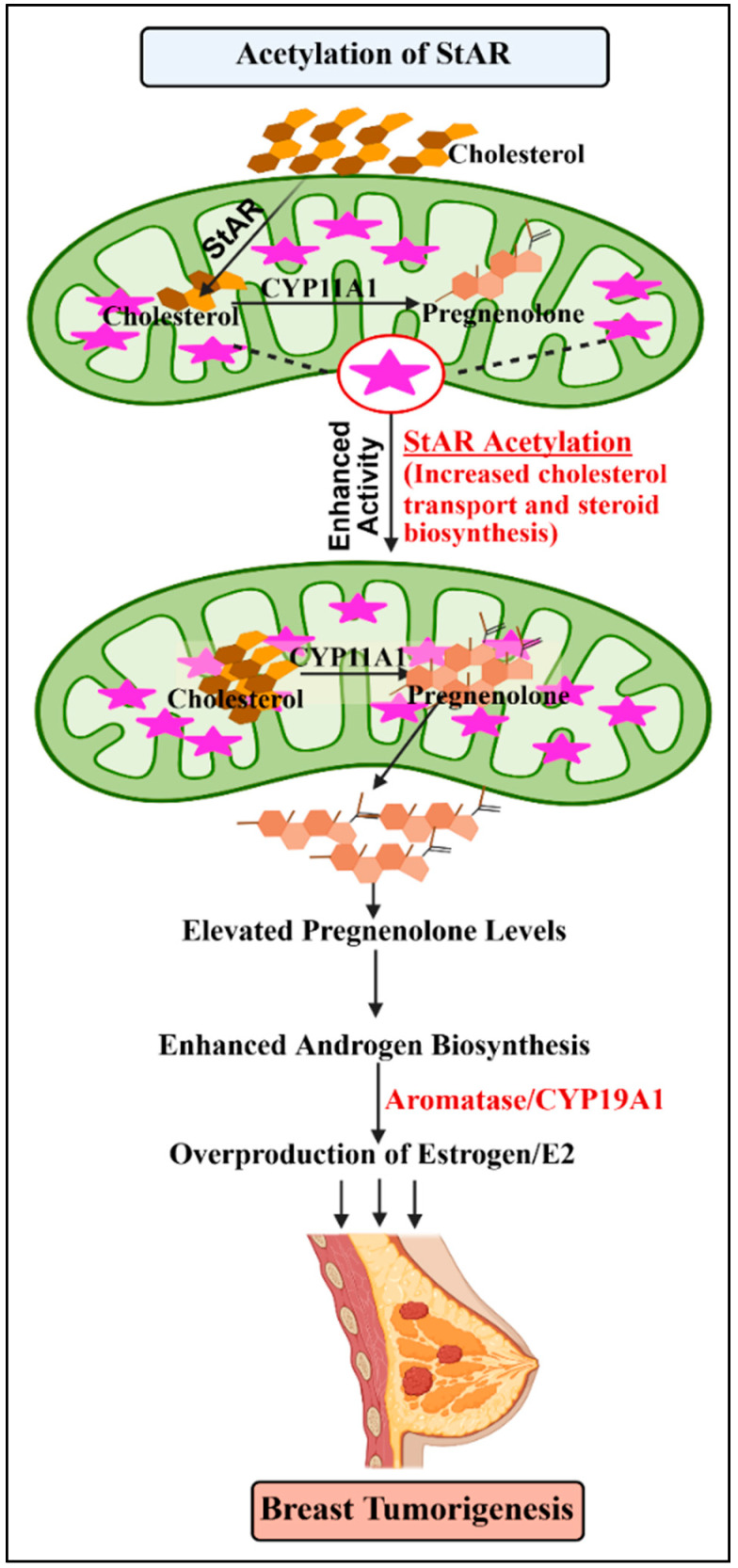
A schematic model illustrating acetylation of StAR in enhancing estrogen/E2 biosynthesis for driving breast pathogenesis. Acetylated StAR promotes increased transport of cholesterol into the mitochondrial inner membrane, where CYP11A1 converts cholesterol into pregnenolone. Elevated pregnenolone biosynthesis leads to increased androgen production, which is subsequently converted to E2 by CYP19A1. The resulting overproduction of E2 activates hormone-dependent signaling, contributing to breast tumorigenesis.

The mechanism accounting for intra-tumoral E2 accumulation, involving StAR acetylation, appears to be a fundamental event in breast tumorigenesis. In various breast cell lines, including MCF12F (non-malignant), MCF7 (HR+), and MDA-MB-231 (TNBC), StAR was identified to be putatively acetylated at eight constitutive lysine (K) residues, i.e., K7, K97, K98, K111, K118, K155, K238, and K253, identified by liquid chromatography–tandem mass spectrometry [8,13]. In addition, StAR was acetylated at K98, K107, K111, and K118 in human H295R adrenocortical cells, suggesting the significance of this novel modification in steroid hormone regulation in pertinent tissues. It is conceivable that StAR lysine residues, recognized in cancerous and non-cancerous breast cells, have discrete effects on E2 levels. Functionally, acetyl-mimetic substitutions (K to glutamine, K→Q) at K111 and K253 have been shown to increase E2 production in both cancerous and non-cancerous breast cells [8,13]. Conversely, deacetylation mimetic events (K→R, arginine) suppressed E2 synthesis, suggesting a potential therapeutic angle in HR+ BC [82]. The characterization of K111 and K253, involving K→Q and K→R mutants, to E2 regulation in mammary tissue, underscores the importance of StAR acetylation in breast physiology and pathology. Therefore, E2-activation, involving aberrant and uncontrolled growth of mammary cells, promotes breast tumorigenesis (Figure 3), in which genetic, epigenetic, and metabolic events play permissible roles.

## 4. Epigenetic Landscape and Its Impact on Breast Tumorigenesis

Epigenetic regulation encompasses heritable changes that impact gene expression without altering the DNA sequence. These include methylation, histone modifications, chromatin remodeling, and non-coding RNA activity, which control diverse activities in normal and diseased states [74,83]. Concerning breast tumorigenesis, epigenetic dysregulation modulates cellular homeostasis, driving malignant transformation. Such changes frequently silence tumor suppressor genes, activate oncogenes, and perturb pathways governing proliferation, apoptosis, and differentiation [83,84]. Age-related epigenetic alterations, including those associated with menopause, may further increase BC susceptibility. Epigenetic modifications are reversible, offering opportunities for early detection and therapeutic intervention [83]. Recent advances in multi-omics technologies have illuminated the complex epigenetic landscape underlying BC pathogenesis [85]. Aberrant activity has emerged as a critical driver of transcriptional reprogramming, with cancer-specific enhancers exhibiting subtype-dependent activity and functional switching during tumor progression. This regulatory plasticity underscores the dynamic nature of enhancer–gene interactions in oncogenesis.

There is increasing evidence that alterations of the epigenetic machinery play a central role in BC. HATs promote transcription by relaxing chromatin structure, whereas HDACs and SIRTs repress gene expression through deacetylation. In BC, certain HATs such as KAT2B and KAT5 exert tumor-suppressive effects, whereas KAT6A, EP300, and SRCs are linked to proliferation, epithelial-to-mesenchymal transition (EMT), and therapy resistance [85,86]. HDACs, particularly Class I HDACs and SIRTs, exhibit context-dependent effects in breast pathology. Whereas HDAC1 and HDAC3 promote proliferation, EMT, and metastasis, HDAC11 and SIRT4 display tumor-suppressive activity, and this dichotomy emphasizes the complexity of targeting HDACs therapeutically [86]. Analyses of TCGA-BRCA RNA-seq data revealed that the majority of HDACs/SIRTs are dysregulated [28]. Consistent with this, many of these epigenetic regulators were mutated or altered in a variety of non-tumorous and tumorous BC cell lines [22,87]. Moreover, knockout of HDAC2 has been shown to inhibit cellular signaling in TNBC cells and alter tumor growth in vivo [88]. However, it was observed that StAR gene transcription was found to be altered in the TCGA-BRCA data cohort compared with normal breast tissue, thereby modulating StAR-driven E2 overproduction in promoting BC [1]. Concomitantly, a link between epigenetic dysregulation and E2 overproduction has been demonstrated [1,28], which unquestionably involves a plethora of processes.

Epigenetic dysregulation contributes to tumor heterogeneity and genomic instability, in which DNA methylation is a critical event. Hypermethylation of tumor suppressor genes impairs DNA and enhances invasiveness [89,90,91]. Genome-wide analyses have identified several methylation-driven genes, including MATK, IFI35, FAM150B, LBXCOR1, WNT10A, and CPT1A, associated with BC [92]. Conversely, hypomethylation can activate oncogenic or immune-modulatory genes such as IL10, NOTCH1, and ABCB1, for promoting tumor progression [93]. Dysregulation of epigenetic modifiers, including DNMT1, DNMT3A/B, and TET1, disrupts methylation dynamics, reinforcing transcriptional reprogramming and genomic instability [93,94]. Emerging evidence implicates that epigenetic alteration in tumor immune evasion is transformed through antigen presentation and interferon signaling [95,96]. Moreover, changes in epigenetic enhancer and super-enhancer landscapes drive oncogene expression and aggressive phenotypes [97]. These multilayered modifications reshape transcriptional and metabolic networks, impacting breast pathogenesis, positioning precision epigenetic intervention for BC therapeutics.

## 5. Overview of AIs in BC Therapy and Endocrine Resistance

Endocrine therapies, involving AIs, are operative in post-menopausal patients; however, their influence beyond several years does not uniformly reduce estrogen/E2-stimulated BC, but rather relapses in certain patient subsets [12]. Preclinical investigations have evaluated the effects of AIs across multiple BC cell lines, demonstrating dose- and time-dependent inhibition of aromatase activity and reduced cell viability [98]. AIs are classified into first-, second-, and third-generation drugs based on their chemical structure, and categorized as steroidal and non-steroidal, and reversible and irreversible groups (Table 1) [12,45,81]. First-generation AIs, including Aminoglutethimide, Fadrazole, and Vorozole, lack aromatase specificity and inhibit other cytochrome P450 enzymes, resulting in glucocorticoid excess and many adverse effects [99]. Their limitations led to the development of second-generation AIs. Accordingly, Formestane, a steroidal AI, showed improved selectivity and greater efficacy as a second-line BC therapy, and fewer side effects than Aminoglutethimide. Overall, second-generation AIs are more potent than first-generation compounds. In addition, Fadrazole, a non-steroidal AI, demonstrated efficacy comparable to Tamoxifen with reduced toxicity, offering an alternative for patients at thromboembolic risk, and achieved a median survival of ~23 months in post-menopausal patients [100]. Third-generation AIs, i.e., Anastrozole, Letrozole, and Exemestane, exhibit higher potency and specificity for aromatase inhibition (Table 1). Steroidal AIs such as Formestane and Exemestane, both derived from androstenedione, irreversibly bind the androgen-binding site of aromatase, acting as pseudo-substrates to suppress E2 synthesis. While third-generation AIs have achieved substantial clinical success with relatively fewer side effects in post-menopausal women, resistance to endocrine therapy remains a significant issue. Endocrine resistance is frequently associated with aggressive phenotypes and poor prognosis, in addition to enhanced mortality. Despite their effectiveness, AIs generate several undesirable long-term side effects, including osteoporosis, breast and vaginal atrophy, depression, diminished libido, and carcinogenesis in some cases in other tissues [1,99]. A recent study has highlighted differences among third-generation AIs; for instance, Exemestane shows comparatively lower disease-free and overall survival than Anastrozole and Letrozole, reflecting variability in clinical outcomes [101].

As stated above, resistance to endocrine therapy for BC treatment remains a major clinical challenge, which can be either de novo, present before treatment, resulting in primary non-responsiveness, or acquired, where tumors initially respond but later become refractory. Moreover, mutation of ER occurs in approximately 20% of patients undergoing endocrine therapy, representing a treatment failure [112]. However, endocrine resistance is not solely driven by alterations in ER [113]. Resistant tumors frequently harbor mutations in genes such as *ESR1*, *PIK3CA*, and *TP53*, alongside defects in DNA repair pathways, structural variations, copy-number alterations, and telomere shortening. These instabilities impair the tumor’s ability to maintain genome integrity, contributing to both primary and acquired resistance. Elucidating these mechanisms provides opportunities for precision-based interventions aimed at improving therapeutic outcomes for HR+ BC [114]. Endocrine resistance to AIs has been discussed in a number of excellent reviews [59,115], and will not be elaborated upon here.

## 6. Therapeutic Targeting of StAR in Hormone-Sensitive BC

It is well-established that BC treatment is contingent on tumor subtypes, TNM stages, HR status, and metastatic burdens [1,60,99,116]. Since overproduction of E2 triggers hormone-sensitive breast tumors, therapeutic strategies for HR+ BC involve suppression of intra-tumoral E2 accumulation. Hormone-dependent BCs generally respond well to endocrine therapies, involving selective ER modulators (SERMs) and AIs, whereas HR- subtypes are frequently unresponsive to SERMs/AIs, resistant, and exhibit higher recurrence rates [1,99,112]. Early-stage breast tumors/cancers are primarily managed surgically, while metastatic BC requires multiple approaches incorporating surgery, chemotherapy, and other treatments. The eventual development of AI resistance can contribute to disease reappearance over time [117]. To mitigate these issues, inhibition of HDACs has emerged as a novel therapeutic strategy targeting StAR, as this cholesterol transporter has been demonstrated to be abundantly expressed in HR+ BC cells compared with their HR- counterparts [2,3,4]. Particularly, HDACIs have been shown to suppress E2 levels, by modulating StAR acetylation patterns, in a variety of HR+ human BC cells and in primary cultures of breast tumor epithelial cells from polyoma middle T-antigen mice. These findings not only open up a new avenue in BC research but also support StAR as a druggable target for suppressing intra-tumoral E2 accumulation for the management of hormone-sensitive BC.

There is increasing evidence that HDACIs exhibit promising results for combating BC, offering limited toxicity and a more favorable safety profile compared to AI-based therapy. HDACIs influence the expression of tumor suppressor genes, oncogenes, and other regulators of cell survival, positioning them as a multifaceted approach in the treatment of HR+ BC [28]. Moreover, HDACIs have been shown to suppress proliferation, induce cell cycle arrest, and promote apoptosis, particularly in models that have developed resistance to endocrine therapy [118]. Mechanistically, HDACIs target multiple oncogenic pathways, including modulation of the Hippo signaling cascade, where activation of tumor-suppressive genes has been associated with improved clinical outcomes. In addition, HDACIs can alter the tumor microenvironment and enhance responsiveness to other systemic treatments, supporting their valuation in combination regimens [119,120]. Regardless of these benefits, off-target effects and toxicities of HDACIs are evident, affecting multiple HDACs and interacting with other proteins, which trigger diverse signaling pathways and reduce therapeutic specificity [121]. Their efficacy as single agents in solid tumors are also limited due to tumor heterogeneity [121].

**Figure 4:**
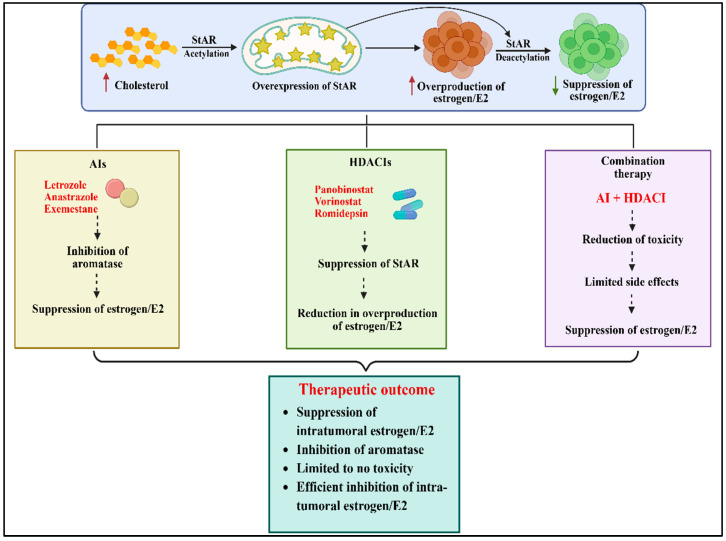
A schematic model elucidating a combination therapy for HR+ BC that targets aromatase and StAR together for suppressing estrogen/E2 accumulation. HDACIs, such as Panobinostat, Vorinostat, and Romidepsin, alter StAR acetylation patterns, resulting in decreased cholesterol transport into the inner mitochondrial membrane, thus reducing estrogen/E2 biosynthesis. Conversely, AIs, such as Letrozole and Anastrozole, by inhibiting aromatase activity and preventing the conversion of androgens to estrogens, diminish E2 levels. A combination therapy with AI and HDACI is expected to improve E2 suppression while reducing toxicity and undesirable side effects, and can be more effective and safer for the management of hormone-dependent BC.

Preclinical evidence has highlighted that three FDA-approved HDACIs alter StAR acetylation patterns, leading to its expression and activity, thus lowering E2 levels in HR+ BC cells [1,2,4]. Epigenetic enzymes have appeared as therapeutic targets for BCs even with challenges [35,122,123] In accordance, Panobinostat has been shown to target key downstream steps and decrease aromatase gene promoters I.3/II that impact BC and inhibits E2 biosynthesis in HR+ MCF7 cells [124]. Since BC is a multifactorial condition, HDACIs can be used in combination with AIs at lower doses, with improved efficacy and limited toxicity, for managing this devastating disease. In a phase I clinical trial, both Panobinostat (an HDAC6 inhibitor) and Letrozole improve the survival of metastatic BC in post-menopausal women [125]. It has been reported that combinatorial effects of Entinostat and Exemestane improve progression free survival in HR+ BC patients [126]. Likewise, an HDACI α-lipoic acid and Exemestane synergistically activate caspase activation and inhibit cell proliferation in HR+ MCF7 and T-47D BC cell lines [127]. We have also found that a combination of HDACIs with Anastrozole reduces StAR-modulated E2 levels in primary cultures of mouse breast tumor epithelial cells [28]. In a phase I clinical trial, both Panobinostat and Letrozole improve overall survival of metastatic BC in post-menopausal women [125]. Therefore, while StAR can be considered as a potential therapeutic target, a combination action of HDACIs and AIs, compared with their monotherapy, is more effective in suppressing intra-tumoral E2 accumulation, in order to alleviate, prevent, and/or treat hormone-responsive BC in women (Figure 4).

## 7. Overdiagnosis and Overtreatment of BC

BC is one of the most prevalent malignant disorders among women globally, in which 1 in 8 women is projected to develop invasive BC during their lifetime, and 1 in 38 dies from the disease [128]. Consequently, while widespread BC screening has increased detection rates, leading to overdiagnosis along with overtreatment occasionally, many of the indolent tumors may never progress to cancers [129]. Estimates of overdiagnosis in contemporary screening vary, ranging from 1% to 54%, reflecting methodological/technological differences [115]. Overdiagnosis refers to the detection of tumors through screening that would not become clinically significant within a patient’s lifetime, often involving slow-growing lesions in older individuals with comorbidities [130,131]. As such, there are challenges for definitive imaging or pathological markers reliably distinguishing benign tumors from cancers, complicating accurate assessment of diagnosis. Despite these concerns, screening remains essential for identifying early-stage lesions, including ductal carcinoma in situ, which carries the potential for progression to devastation [131].

Overtreatment frequently follows overdiagnosis and includes surgery, radiation, chemotherapy, and/or endocrine therapy [132,133]. Such interventions can result in various consequences, including morbidity, lymphedema after axillary dissection, cardiotoxicity associated with certain chemotherapies, chronic fatigue, and declines in physical function particularly in older women with low risks [132]. Moreover, extended use of AIs contributes to treatment-related burdens. While effective in suppressing estrogen/E2 for HR+ breast tumors, prolonged AI therapy induces systemic hypo-estrogenism, leading to osteoporosis, arthralgia, cardiovascular complications, and neuropsychological symptoms [1]. Additionally, sustained estrogen deprivation may promote acquired resistance through *ESR1* mutations or activation of compensatory signaling pathways, necessitating therapeutic escalation and compounding toxicity. Epigenetic reprogramming signifies a promising alternative to overcome these situations, as HDACIs not only act at multiple levels, but also mitigate limitations associated with prolonged endocrine therapy.

## 8. Inhibition of HDACs for BC Therapeutics

A large body of evidence indicates that HDACs are either overexpressed or mutated in BC and other cancers, leading to chromatin hypoacetylation and transcriptional repression, which are associated with poor clinical outcomes [134,135]. In addition, HDACIs have been shown to modulate acetylation of StAR and aromatase, reactivate gene expression, and reduce viability and promote apoptosis in cancer cells. The clinical prospects of HDACIs in BC treatment, as well as overcoming resistance, are emerging. For example, Entinostat has been shown to inhibit tumor-initiating cells (TICs), which are closely linked to metastasis and therapy resistance [136]. It also reverses the EMT and reduces TIC proliferation in TNBC. A phase II clinical trial with 43 patients having HR+ metastatic BC treated with a combination of Tamoxifen and Vorinostat showed over 40% tumor reduction [119]. Treatment with PCI-24781, an HDACI, successfully re-sensitized these tumors to Tamoxifen by lowering Bcl-2 and AKT signalling, thereby contributing to cancer cell death [1,137]. It has been reported that SIRT1, a class III HDACI, plays a pivotal role in regulating the expression and activity of aromatase in BC cells [20,60]. Moreover, pharmacological inhibition of SIRTs has been shown to reduce aromatase mRNA and protein levels in both hormone-sensitive MCF-7 and TNBC (MDA-MB-231) cells. The anti-tumorigenic effects of HDACIs are partly attributed to their ability to overcome endocrine therapy resistance, particularly in patients with metastatic BC [1,28]. Panobinostat has been demonstrated to enhance histone modification in H3 and H4, induce apoptosis, reduce cell proliferation, and reactivate ER expression in TNBC cells [124]. It has also exhibited resistance by suppressing NF-κB1 expression in AI-resistant BC cells [1,138]. In support of these data, we reported that FDA-approved HDACIs, including Panobinostat and SAHA, alter StAR acetylation patterns and suppress E2 levels in human and mouse HR+ BC cells [2].

While HDACIs display favorable outcomes in BC treatment by modulating the epigenetic landscape, reactivating tumor suppressor genes, and inhibiting oncogenic pathways, their clinical impact faces several challenges. Studies have reported that HDACI monotherapy in breast tumors results in limited responses in phase II trials [139,140]. Moreover, epigenetic plasticity allows cancer cells to bypass HDAC inhibition or activate alternative survival pathways [141]. Many HDACIs lack isoform selectivity and also target non-histone substrates, leading to toxicity, off-target and limiting achievable doses [121,140]. Table 2 shows a list of both FDA-approved and many clinical-phase trial HDACIs that are tested for the treatment of hormone-sensitive BC in diverse situations, in which the clinical efficiencies of many of these HDACIs are limited with the outcomes.

## 9. Development of a Combination Therapy with AI and HDACI for Mitigating BC

A variety of combination therapies have been evaluated to enhance the efficacy of endocrine therapy in hormone-responsive BC (Table 3). Preclinical studies have demonstrated synergistic interactions between AIs and other target agents to improve the clinical outcomes. For instance, cyclooxygenase-2 (COX-2) inhibitors have been combined with AIs, as inhibition of COX-2 reduces aromatase activity, further suppressing estrogen synthesis [154,155]. Similarly, CDK4/6 inhibitors with AIs have demonstrated improved efficacy in HR+/HER2- BC [156]. Moreover, suppression of autophagy, a cellular survival mechanism, has been shown to sensitize BC resistant cells to AI treatment, supporting combination strategies incorporating autophagy inhibitors to overcome therapeutic resistance [98]. Concurrently, the combined effects of HDACIs such as Panobinostat and Vorinostat, PARP inhibitors (e.g., Talazoparib and Olaparib), and the hypomethylating agent Decitabine have shown benefits in both BC and ovarian cancer models [157]. These combinations simultaneously target epigenetic regulation and DNA repair pathways, resulting in decreased expression of proteins involved in chromatin remodeling and DNA damage. The effectiveness of PARP inhibitor-HDACI combinations has been shown to depend on BRCA mutations and tumor microenvironment, highlighting the importance of precision-guided approaches [157]. HDACIs are increasingly being explored in multi-target and dual-inhibition strategies to overcome resistance and enhance therapeutic efficacy in various BC stages [158]. By combining BET bromodomain proteins, DNMTs, lysine-specific demethylase 1, and G-quadruplex structures, with HDACIs, a number of studies have demonstrated a synergistic antitumor effect in multiple BC models [159,160,161,162]. These properties include suppression of transcriptional programs, reactivation of silenced tumor-suppressor genes, inhibition of cancer stem cell maintenance, and disruption of telomere stability, with particularly pronounced efficacy in aggressive BC and TNBC models. Beyond epigenetic modulation, HDACIs have been paired with key oncogenic enzymes, such as PI3K and Janus kinase–signal transducer and activator of transcription, to target critical proliferation and survival signaling pathways. Specifically, the combination of PI3K-HDACI not only induces cell-cycle arrest and apoptosis but also sensitizes tumors to extrinsic apoptotic signals and impairs tumor growth across multiple BC subtypes [163,164].

**Figure 5:**
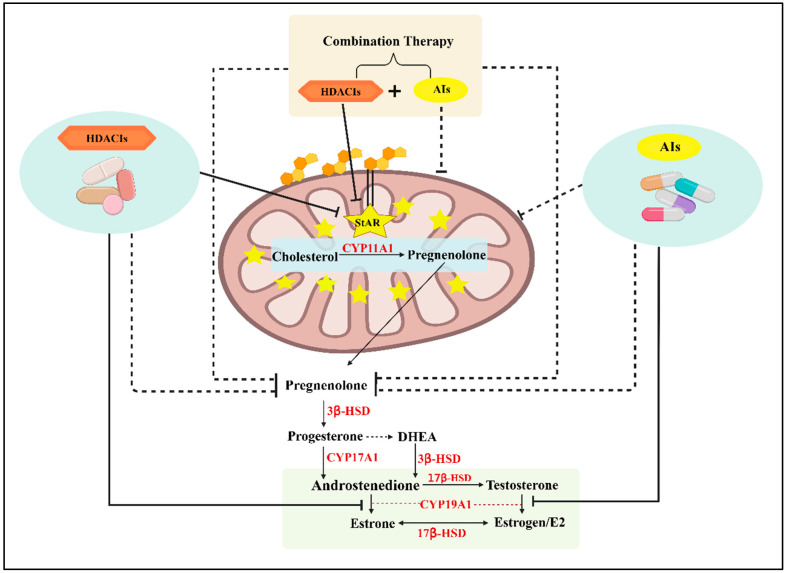
A potential combination therapeutic approach, involving AIs and HDACI, at suboptimal doses for HR+ BC. AIs block the aromatase enzyme activity and inhibit estrogen/E2 production. HDACIs reduce E2 synthesis by downregulating the function of StAR, involving acetylation, along with cholesterol transport. The bottom panel illustrates the estrogen biosynthetic pathway. A combination therapy is expected to effectively suppress intra-tumoral E2 accumulation, reduce toxicity, and limit endocrine resistance, offering a promising new approach for alleviating hormone-dependent BC.

In HR+/HER2- BC, a CDK inhibitor, Palbociclib, in the PROMETEO II trial showed that combining Palbociclib with Letrozole after neoadjuvant chemotherapy induced cell cycle arrest in the majority of patients [165]. Similar effects were seen in treatment with Ribociclib in combination with Letrozole [166]. Furthermore, using monoclonal antibodies, Pertuzumab, Trastuzumab, and Docetaxel showed substantial improvement in overall survival of patients affected with HER2+ BC metastasis [170]. Therefore, these therapeutic approaches in combination provide a rational and pharmacological framework to improve clinical outcomes, delay resistance, and target multiple pathways for BC prognosis and treatment. For hormone-sensitive BC, HDACIs modulate endocrine therapy efficacy through ERα-targeted strategies, including SERD/SERM–HDAC hybrids, as well as Tamoxifen combined with Vorinostat, which promote ERα degradation and epigenetic silencing of estrogen-responsive genes [4,177]. This dual approach simultaneously impacts hormone receptor signaling and E2 sensitization, resulting in anti-proliferative activity, improved tumor suppression, and the potential to overcome resistance compared with conventional endocrine therapies [2,4]. TCGA-BRCA data revealed that steroidogenic enzymes and steroid hormone-related genes such as StAR, STARD3, CYP11A1, 17βHSD, 3βHSD and CYP19A1 are abundantly expressed in cancerous breast tissues compared with their non-cancerous counterparts, which may result in overproduction of estrogen/E2 in luminal BC subtypes [2,4,22,178,179]. However, an overlap between AIs and HDACIs pathways is evident as they suppress E2 biosynthesis by targeting aromatase and StAR, respectively, and can act synergistically in combating HR+ BC. Based on these data, we envision that a combinatorial approach, involving AIs and HDACIs, at their suboptimal doses, is effective for suppression of estrogen/E2 accumulation for hormone-responsive BC while reducing the toxicity and adverse side effects for prolonged therapies (Figure 5). This strategy offers multiple advantages, including attenuation of intra-tumoral estrogen/E2, prevention or delayed endocrine resistance, decreased tumor proliferation, and improved treatment tolerability with limited undesirable side effects.

## 10. Conclusions

Dysregulation of androgen and estrogen biosynthesis has been primarily linked to the pathogenesis of HR+ BC [1,2,28,60]. It is unambiguous that long-term exposure of breast tissue to estrogen/E2, accompanied by elevated genotoxic and oncogenic signaling, is an essential event in BC risk and incidence. While endocrine therapies, involving AIs, are the mainstay of BC treatment in post-menopausal women, AIs suppress whole body estrogen levels and generate undesirable long-term side effects, including resistance, which is the leading cause of cancer death [1,45,47,99,107,112,180]. Emerging evidence indicates that HDACIs are not only clinically efficacious, safe, and display limited toxicity in comparison to AIs against multiple oncogenic events, but they also display favorable outcomes in numerous aspects in cancer cells (reviewed in References [1,113,180]). Concurrently, we reported that steroid hormone regulator StAR is a novel acetylated protein, along with the identification of several lysine residues undergoing acetylation and deacetylation, providing mechanistic insights into the influence of this novel PTM in E2 regulation in mammary tissue. In addition, a variety of HDACIs, including FDA-approved ones, by altering StAR acetylation patterns, suppress E2 accumulation in hormone-sensitive human and mouse BC cells/tissues [1,2,4], highlighting the therapeutic relevance of StAR for HR+ BC. While the involvement of AIs and HDACIs, individually, has been studied in BC treatment, both generate, especially AIs, diverse antagonistic effects. Therefore, a combination of AIs and HDACIs, at their suboptimal doses, could be a novel and promising therapeutic target for combating hormone-responsive BC. AIs block the enzymatic conversion of androgens to estrogens by inhibiting the aromatase activity. HDACIs modulate StAR acetylation, thereby impairing E2 biosynthesis [4]. These complementary actions, involving diminished aromatase activity by AIs and decreased StAR function by HDACIs, are instrumental for efficient diminution of intra-tumoral E2 accumulation, benefiting millions of women who are afflicted with this life-threatening disease. Such regimens could not only mitigate long-term adverse side effects but also delay resistance while maintaining efficacy in HR+ tumors where both aromatase and StAR are overexpressed [8,10]. Future directions should include studies elucidating the HDACI-AI interplay and biomarker-guided patient stratification. These efforts could establish AI- and HDACI-based combination therapy, positioning to offer new druggable targets, for effective suppression of intra-tumoral estrogen/E2 accumulation in improving both survival and quality of life in women who are afflicted with hormone-sensitive BC.

## Figures and Tables

**Table 1:** A list of first-, second-, and third-generation AIs that are used frequently in the clinics for the treatment and/or management of BC.

Drugs	Types	Drug Information	Targets	Brand Names/ Companies	References
Anastrozole	Non-steroidal AI	3rd generation AI (FDA-approved)	Adjuvant therapy for pre- and post-menopausal HR^+^ BC and metastatic HR^+^ BC	Arimidex/AstraZeneca	[102]
Letrozole	Non-steroidal AI	3rd generation AI (FDA-approved)	Adjuvant therapy in post-menopausal women	Femara/Teva	[103,104]
Exemestane	Steroidal AI	3rd generation AI (FDA-approved)	Used often after Tamoxifen for BC	Aromasin/Pfizer	[105,106]
Formestane	Steroidal AI	2nd generation AI	Second-line therapy for BC	Lentaron/Novartis	[107,108]
Fadrazole	Non-steroidal AI	2nd generation AI	Second-line therapy for BC	Afema/Novartis	
Aminoglutethimide	Non-steroidal AI	1st generation AI	First-line therapy for BC	Elipten, Cytadren, Orimeten, and others/Willow Birch Pharma	[109]
Tamoxifen	SERM (anti-estrogen)	FDA-approved	Adjuvant therapy for pre-, post-menopausal, and metastatic HR^+^ BC; first-line therapy for metastatic BC	Nolvadex, Solmatox/AstraZeneca	[110,111]

**Table 2:** A list of a variety of HDACIs that are used in BC treatment and/or are under evaluation in various clinical trials.

Drugs	HDAC Classes	Drug Status	Tumor Types	Companies	References
Entinostat (MS-275)	Class I, IIa	Phase III clinical trial	BC metastasis	Syndax Pharmaceutical, Inc., National Cancer Institute	[142,143]
Tinostamustine (EDO-S101)	HDACI + alkylating agent	Phase I/II	BC, hematologic malignancy	Mundipharma	[144]
Vorinostat (SAHA)	HDAC pan-inhibitor	Phase II	BC metastasis	Zolinza/Merk	[145]
Panobinostat	HDACIs	Phase I	BC metastasis	Alliance for Clinical Trial Oncology	[125]
Pracinostat (SB939)	HDACIs	Preclinical	BC metastasis	Ningxia Medical University	[146]
Romidepsin	HDACIs combination therapy	Phase I/II	TNBC or BC metastatic	University of Kansas Medical Center	[147]
Mocetinostat (MGCD0103)	Class I/IV	Phase II	BC metastatic	Mirati Therapeutics	[148,149]
Belinostat (Beleodaq)	HDACIs	Preclinical	BC metastasis	Taipei Medical University	[150]
Trichostatin A	Class I/II	Preclinical	BC metastasis	Sichuan Cancer Hospital and Institute	[151]
Ricolinostat	HDAC6I	Phase I	BC metastasis	Columbia University	[152]
CXD101	Class I selective	Phase I/II	Colorectal and other cancers	Celleron Therapeutics	[153]

**Table 3:** A list of drugs and/or inhibitors that have been assessed as a combination therapy for combating a variety of BCs.

Drug Combinations	Clinical Outcomes	Mechanistic Assessment	Drug Status	References
Palbociclib + letrozole	HR+/HER2− metastasis	CDK4/6 inhibition + estrogen deprivation	FDA-approved	[165]
Ribociclib + Letrozole	HR+/HER2− metastasis	CDK4/6 blockade	FDA-approved	[166]
Abemaciclib +Fulvestrant	HR+/HER2− metastasis	CDK4/6 inhibition + ER degradation	FDA-approved	[167]
Everolimus + Exemestane	HR+ metastasis	mTOR inhibition	FDA-approved	[168]
Alpelisib + Fulvestrant	HR+/HER2−, PIK3CA-mutant	PI3Kα inhibition	FDA-approved	[169]
Trastuzumab + Pertuzumab + Docetaxel	HER2+ metastasis	Dual HER2 blockade and chemotherapy synergy	Phase III	[170]
Lapatinib + Trastuzumab	HER2+	Vertical HER2 pathway inhibition	Phase III	[171]
Atezolizumab + Nab-Paclitaxel	TNBC metastasis	Immune checkpoint inhibition and chemo-induced immunogenicity	Previously approved	[172]
Olaparib monotherapy + chemotherapy	BRCA mutation-related BC	PARP inhibition	Approved as monotherapy	[173,174]
Tamoxifen + Vorinostat (SAHA)	HR+ BC	ER signalling	Phase II	[175]
Pembrolizumab + chemotherapy	TNBC metastasis	PD-1 inhibition	FDA- approved	[176]

## Data Availability

Data reported in this study are included in this manuscript.
